# Sample size, sample size planning, and the impact of study context: systematic review and recommendations by the example of psychological depression treatment

**DOI:** 10.1017/S003329172100129X

**Published:** 2021-04

**Authors:** Raphael Schuster, Tim Kaiser, Yannik Terhorst, Eva Maria Messner, Lucia-Maria Strohmeier, Anton-Rupert Laireiter

**Affiliations:** 1 Department of Psychology, University of Salzburg, Austria; 2 Center for Clinical Psychology, Psychotherapy and Health Psychology, University of Salzburg, Austria; 3 Department of Psychology, University of Greifswald, Germany; 4 Department of Clinical Psychology and Psychotherapy, University of Ulm, Germany; 5 Department of Research Methods, University of Ulm, Germany; 6 Faculty of Psychology, University of Vienna, Austria

**Keywords:** Depression, digital psychiatry, sample size calculation, statistical power, study design, trial pre-registration

## Abstract

**Background:**

Sample size planning (SSP) is vital for efficient studies that yield reliable outcomes. Hence, guidelines, emphasize the importance of SSP. The present study investigates the practice of SSP in current trials for depression.

**Methods:**

Seventy-eight randomized controlled trials published between 2013 and 2017 were examined. Impact of study design (e.g. number of randomized conditions) and study context (e.g. funding) on sample size was analyzed using multiple regression.

**Results:**

Overall, sample size during pre-registration, during SSP, and in published articles was highly correlated (*r*'s ≥ 0.887). Simultaneously, only 7–18% of explained variance related to study design (*p* = 0.055–0.155). This proportion increased to 30–42% by adding study context (*p* = 0.002–0.005). The median sample size was *N* = 106, with higher numbers for internet interventions (*N =* 181; *p* = 0.021) compared to face-to-face therapy. In total, 59% of studies included SSP, with 28% providing basic determinants and 8–10% providing information for comprehensible SSP. Expected effect sizes exhibited a sharp peak at *d* = 0.5. Depending on the definition, 10.2–20.4% implemented intense assessment to improve statistical power.

**Conclusions:**

Findings suggest that investigators achieve their determined sample size and pre-registration rates are increasing. During study planning, however, study context appears more important than study design. Study context, therefore, needs to be emphasized in the present discussion, as it can help understand the relatively stable trial numbers of the past decades. Acknowledging this situation, indications exist that digital psychiatry (e.g. Internet interventions or intense assessment) can help to mitigate the challenge of underpowered studies. The article includes a short guide for efficient study planning.

## Introduction

Statistical power is the probability to detect the effect one is looking for, given the effect exists. Hence, sufficient statistical power is a key criterion for studies based on inference statistics. Considering the convention for statistical power (preferably *>*80%), the fields of psychology and neuroscience suffer from a considerable lack of adequately powered studies (Szucs & Ioannidis, 2017). For mental health, a comprehensive review of clinical trials registered in ClinicalTrials.gov (*N* = 96 346) revealed a modest median sample size of 61 patients per study (Califf et al., 2012) which restricts sensitivity to detect treatment effects and impedes many relevant analyses (e.g. moderate between-group effect sizes caused by desirable active control group designs, or moderator analyses). It is therefore important to understand the process and influencing factors of sample planning in clinical research.

According to Altman and Simera's history of the evolution of guidelines, critically small sample sizes were mentioned as early as in the first part of the twentieth century (Altman & Simera, 2016). Over time, increasing awareness about the importance of sample size and sample size planning (SSP) has led to the development of recommendations for SSP (cf. Appelbaum et al., 2018). For randomized controlled trials (RCTs), the CONSORT 2010 guidelines (Moher et al., 2012) include the following statement:

*Authors should indicate how the sample size was determined. […] Authors should identify the primary outcome on which the calculation was based […], all the quantities used in the calculation, and the resulting target sample size […]. It is preferable to quote the expected result in the control group and the difference between the groups one would not like to overlook. […] Details should be given of any allowance made for attrition or non-compliance during the study*.

These recommendations reassemble the most important statistical SSP determinants for RCTs. Box 1 features the relevant parameters for comprehensible SSP, which aims at providing the reader with all necessary information to assess the full process of sample size determination. From a mathematical perspective, those determinants suffice to estimate the required sample size of a given trial. Sample requirements will be higher with a lower *α* level and with a higher *β* level (study power). The smaller the expected treatment effect (e.g. using an active control condition) and the correlation among repeated measures (e.g. caused by a therapeutic change or long time intervals), the higher is the required sample size. Equally, more study conditions and higher dropout will increase demand for participants.

Box 1. Statistical sample size determinants for randomized clinical trials.

**Table:** 

(1) Applied statistical test (e.g. ANCOVA or hierarchical model) (2) *α* level (probability of Type I error, conventionally set to ≤5%) (3) *β* level (probability of Type II error, conventionally set to ≥80%) (4) Statistical power (complement of a Type II error, 1 − *β*) (5) Number of trial conditions (6) Expected treatment effect (e.g. incidence, or effect size) (7) Number of repeated measures (8) Correlation among repeated measures (9) Expected dropout

Besides those statistical determinants that are based on study design, however, it is easy to imagine that study context influences the SSP process. Relevant factors typically relate to the practical feasibility of a study. For example, funding has repeatedly been shown to impact SSP parameters, such as sample size or reported effect sizes in medicine and psychiatry (Falk Delgado & Falk Delgado, 2017; Kelly et al., 2006). Additionally, the ease of access to patient populations and sufficient resources constitute important factors (Dattalo, 2008). At this, efficacy trials are frequently conducted as pilot studies, while effectiveness trials usually reflect later stages of research in routine care. As the last example, the provision of treatment in psychiatric care is costly, as many interventions are resource-intense and exhibit limited scalability (e.g. psychological treatment). In this regard, Internet interventions are increasingly recognized as a useful vehicle in mental health research (Domhardt, Cuijpers, Ebert, & Baumeister, 2021), and their efficient application leads to expectably larger sample sizes (Andersson, 2018).

Considering this situation, SSP at times appears to be in a dilemma. On one side, statistical inference requires sufficiently large sample sizes to produce reliable outcomes. On the other side, practical restrictions, such as limited patient access, and financial resources constrain study designs. Even though some researchers argue that statistically underpowered studies do constitute a negligible problem, because individual findings could, anyways, be accumulated into meta-analytic evidence, other groups criticize this view due to the risk of introducing bias (e.g. file drawer problem, or biased estimates of treatment effects), in case a relevant proportion of studies do not find their way into the analysis (Califf et al., 2012; Tackett, Brandes, King, & Markon, 2019; Wampold et al., 2017). To mitigate the risk of accumulating bias, the CONSORT guideline stresses the importance of unbiased, properly reported studies that need to be published irrespective of their results (Moher et al., 2012). Trial pre-registration and registered reports can be seen as an increasingly recognized strategy towards such scholarly reporting practices.

Taken together, the topic of SSP (and achieved sample size) has been discussed for several decades. Previous studies have shown that current psychological and psychiatric research still suffers from low sample sizes, which can influence meta-analytic evidence or the knowledge gain from individual trials. It is therefore important to understand the factors that influence how researchers plan their sample sizes.

The present study investigated the extent to which guideline recommendations were implemented into SSP for RCTs on interventions for depression. Depression was chosen as it is one of the most relevant common mental health disorders to date, and, therefore, also constitutes a frequently investigated disorder. On a descriptive level, the provision of SSP is presented together with pre-registered and achieved sample size. In a second step, study design (e.g. number of study arms and number of repeated measures) was tested together with study context factors (e.g. funding, routine setting) to quantify their influence on actually achieved sample size. More explicitly, we were interested in the following questions: What information about SSP do studies provide? Do studies attain their pre-registered sample size? To which proportion does study design influence sample size? To which proportion does study context influence sample size? To support efforts of adequate SSP, recommendations are provided in Appendix 1.

## Methods

### Literature selection

We selected studies from a current meta-analysis that investigated the effects of psychological treatment for adult major depression (Cuijpers, Karyotaki, Reijnders, & Ebert, 2019). In this study, a database of randomized trials from 1966 until 2017 provided the primary literature. This database has been described in a methods paper earlier (Cuijpers, van Straten, Warmerdam, & Andersson, 2008). In short, the database draws on the bibliographical databases PsycINFO, PubMed, Embase, and Cochrane Central Register of Controlled Trials and is being updated every year. Since the present study focuses on current SSP practices, we only included studies between 2013 and 2017.

### Quality assessment and data extraction

We relied on quality ratings provided in the principal study. This previous quality assessment was based on four selected criteria of the Cochrane risk of bias assessment tool (Higgins et al., 2011). The applied criteria included the following: generation of allocation sequence, allocation concealment, masking of assessors, and handling of incomplete outcome data (e.g. intention-to-treat analyses). Principal ratings were conducted by two independent researchers, who solved eventual disagreements by discussion.

For the present study, data extraction was protocol-based (structured guide of 24 items) and was conducted in dyads by RS, TK, YT, EM, and two research assistants. The protocol entailed general items (e.g. publication year, achieved sample size, and calculated sample size), as well as basic SSP variables (number of study groups, type of control group, and number of repeated measurements), and several items for comprehensible SSP (e.g. type of statistical test, effect size, the justification for effect size, correlation of repeated measures, or expected dropout). We decided to analyze studies between 2013 and 2017, as our priority was to provide information on the current conduct of SSP. Assessment of planned sample size was based on the first available entry of the pre-registration history. The type of control group was coded as ‘passive CG’ whenever the study did not include any active comparator.

### Data analysis

Data were analyzed using descriptive and inferential statistics. Descriptive statistics were used to depict the frequency and quality of SSP in current depression trials. Whenever applicable, bias-corrected and accelerated (BCa) 95% confidence intervals (CIs) were calculated. The three dependent interval variables ‘achieved sample size,’ ‘calculated sample size,’ and ‘pre-registered sample size’ were positively skewed, and, therefore, transformed logarithmically (cf. Appendix 1). All further requirements for *t* tests and linear regression were checked before analysis. Non-parametric tests were applied whenever requirements for parametric analysis were violated.

Multiple regression was used to predict the dependent variable ‘achieved sample size.’ The sensitivity of regression analyses (deviation from zero in a fixed model) was calculated using G*Power (Faul, Erdfelder, Buchner, & Lang, 2009). For the model incorporating the three basic SSP variables (number of conditions, type of control group, number of repeated measures between pre- and post-assessment) in *k* = 78 studies with 80% power, sensitivity was *f*^2^ = 0.145 (*R*^2^ = 0.11, or 11% of variance). The full regression model incorporated four additional context variables: treatment modality (face-to-face/online), setting (effectiveness/efficacy), funding (yes/no), and pre-registration (yes/no). This model resulted in a sensitivity of *f*^2^ = 0.211 (*R*^2^ = 0.16, or 16% of variance). For the group of studies that included sample size calculation (*k* = 44), a second regression was carried out. This statistical model resulted in a sensitivity of *f*^2^ = 0.273 (*R*^2^ = 0.21, or 21% of variance) for the three SSP predictors and *f*^2^ = 0.393 (*R*^2^ = 0.28, or 28% of variance) for the full model.

### Selection of included studies

The present study is based on selected studies from a previous meta-analysis investigating the effects of psychological treatments for depression (Cuijpers et al., 2008) in a sample of *k* = 289 clinical trials. All studies of the principal meta-analysis that had been published between 2013 and 2017 were included in the analysis, resulting in a sample of *k* = 89 primary studies. Of those studies, a proportion of *k* = 11 studies (14%) was excluded. Reasons for exclusion from the analysis were as follows: not published in a peer-reviewed journal (one article), peer-to-peer treatment (two articles), depression not the primary psychiatric outcome (two articles), subclinical sample (two articles), prevention in a student sample (one article), letter to the editor (one article), actually published before 2013 (one article), and analysis limited to descriptive statistics only (one article).

## Results

### Characteristics of included studies

**Fig. 1.**
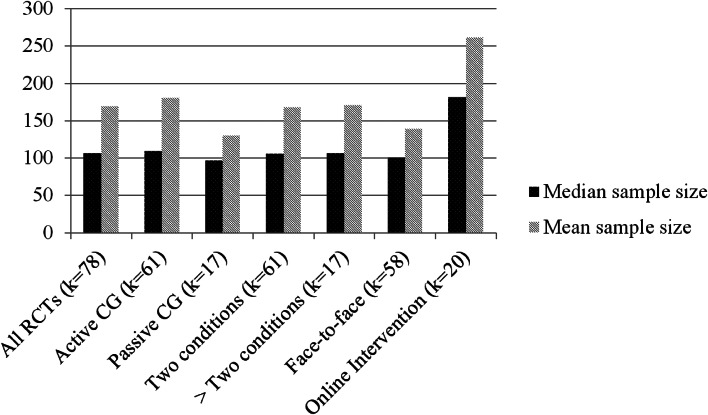
Achieved sample size and its (missing) relation to study design. Conversely, sample sizes of Internet interventions exceed those of face-to-face therapy by around 80%, which underlines the relevancy of digital psychiatry to address the issue of low statistical power in clinical research.

Table 1 presents the characteristics of included studies and Fig. 1 depicts the relationship between study design and achieved sample size. Of the analyzed studies, 70.0% implemented intention-to-treat analysis, 15.5% implemented per-protocol analysis, and 8.6% implemented both, resulting in 5.9% unspecified analysis. The following statistical test(s) were applied during principal analysis: linear mixed models (39.6%), analysis of covariance (ANCOVA) (31%), analysis of variance (ANOVA) (13.8), regression (10.4%), χ^2^ test (12.1%), and *t* test (19.0%).

**Table 1.** Characteristics of included studies

Analyzed studies (%)	78 (100)
Pre-registered studies (%)	46 (59.0)
Median sample size	106
Study context	
- Efficacy trial (%)	33 (42.3)
- Effectiveness trial (%)	44 (56.4)
- Unclear	1 (1.3)
Setting	
- Face-to-face (%)	57 (73.1)
- Internet intervention (%)	20 (25.6)
- Blended (%)	1 (1.3)

### Analysis of SSP determinants

**Fig. 2.**
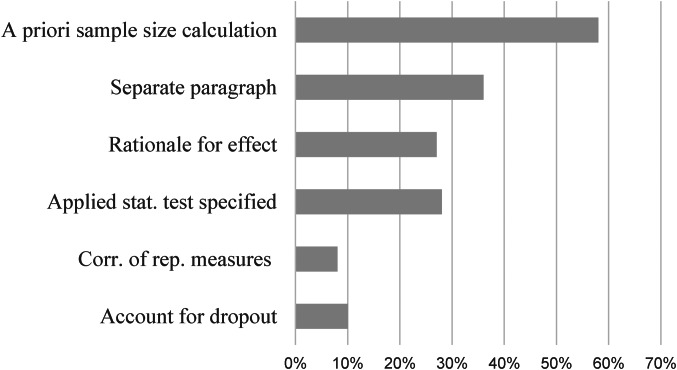
Provision of sample size determinants in current trials on depression; % = percent; *k* = number of studies. Note that only a small fraction of trials provide sufficient information for comprehensible SSP. About one-third provides information on basic SSP determinants.

Most studies followed the significant convention of *α* = 5% together with power = 80%. On average, a treatment effect of *d* = 0.52 (s.d. = 0.17; CI: 0.46–0.59) was implemented, which did not differ by type of control group (*x*^2^_(1,23)_ = 1.26; *p* = 0.205), nor setting (*x*^2^_(1,23)_ = 0.525; *p* = 0.600). Expected treatment effects were not normally distributed, but instead exhibited clear kurtosis around *d* = 0.5 (64% + −0.1; cf. Appendix 1). The remaining determinants for comprehensive SSP are presented in Table 2. Figure 2 depicts the proportions of studies providing information for comprehensible SSP.

**Table 2.** Determinants of comprehensive sample size planning

*α* level	*n* (%)
5%	36 (46.2)
*<*5%	4 (5.1)
no information	38 (48.7)
*β* level	
80%	33 (42.3)
*>*80%	10 (12.8)
no information	35 (44.9)
*N* conditions	
2 (%)	61 (78.2)
3 (%)	14 (17.9)
≥4 (%)	3 (3.9)
Number of repeated measurements^a^	
2 (%)	62 (79.5)
3–4 (%)	8 (10.2)
5–12 (%)	8 (10.2)
Correlation among repeated measures (s.d. of *r*)	0.25 (0.26)
Expected treatment effect (Cohen's *d;* s.d. of *d*)	0.52 (0.17)
Subgroup analysis conducted	31 (53.4)
Actual study dropout	
Face-to-face (% of *N*)	29 (21)
Internet intervention (% of *N*)	51 (19.9)

a Between pre- and post-assessment.

### Effect of study design and study context on the sample size

**Fig. 3.**
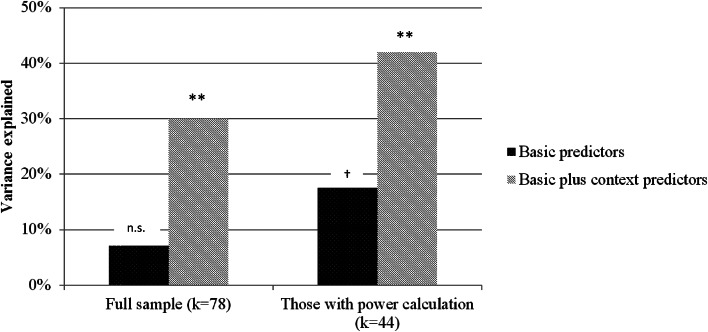
Explained variance (of sample size) of three important SSP determinants, compared to a regression model implementing those predictors together with four study context variables (cf. Table 3); ** *<*0.01; † = 0.055; *k* = number of studies.

This section quantified the extent to which the study design predicted achieved sample size. In two consecutive steps, three SSP determinants and four study context factors were implemented into a block multiple linear regression model to estimate their impact on sample size. Regression models were estimated for the full sample, as well as for studies featuring at least some form of SSP in their articles. For the full sample, the three basic predictors did not explain significantly more variance than the null model (*R*^2^ = 0.07, *F*_(3,72)_ = 1.79, *p* = 0.155). When context factors were added, the regression explained a significant proportion of variance (*R*^2^ = 0.30, *F*_(7,68)_ = 3.59, *p* = 0.002). A comparable pattern with higher proportions of explained variance emerged for those studies with SSP (Block 1: *R*^2^ = 0.18, *F*_(3,39)_ = 2.75, *p* = 0.055; Block 2: *R*^2^ = 0.42, *F*_(7,35)_ = 3.60, *p* = 0.005). Figure 3 depicts the proportions of explained variance for both regressions in the full sample and the SSP sample. Details on standardized regression coefficients are presented in Table 3. Furthermore, sample sizes in pre-registration corresponded to a very high extend with sample requirements during power calculation (*r* = 0.887, *p* *<* 0.001). An even higher correlation was found between sample size in power calculation and achieved sample size (*r* = 0.954, *p* *<* 0.001).

**Table 3.** Predictive value of study design (SSP determinants), and study design plus study context variables for the dependent variable achieved sample size

SSP determinants	Full sample (*k* = 78)			Power calculation only (*k* = 44)		
	*β*	*t*	*p*	*β*	*t*	*p*
Constant		16.07	0.00		12.31	0.00
Number of conditions	0.07	0.56	0.58	0.23	1.50	0.14
Type of control group (0 = active)	−0.09	−0.77	0.45	−0.02	−0.16	0.87
Number of repeated measures	−0.23	−1.96	0.05	−0.30	−2.00	0.05
SSP determinants plus study context	*β*	*t*	*p*	*β*	*t*	*p*
Constant		12.95	0.00		9.56	0.00
Number of conditions	0.12	1.11	0.27	0.38	2.61	0.01
Type of control group (0 = active)	−0.11	−1.07	0.29	0.01	0.06	0.95
Number of repeated measures	−0.27	−2.51	0.01	−0.26	−1.81	0.08
Setting (0 = face to face)	0.30	2.73	0.01	0.20	1.44	0.16
Pre-registered trial (0 = yes)	−0.11	−0.97	0.34	−0.26	−1.91	0.06
Care-context (0 = effectiveness)	−0.28	−2.49	0.02	−0.34	−2.32	0.03
Funding (0 = yes)	−0.15	−1.37	0.18	−0.15	−1.03	0.31

Note. *k* = number of studies.

## Discussion

Aiming to investigate the conduct of SSP, this article analyzed 78 RCTs on psychological treatment for major depression. Besides providing information on pre-registered and achieved sample size, the article estimated the impact of study design and study context on sample size.

Principal findings indicate that the average RCT for depression includes around 100 patients. Furthermore, there was striking concordance between pre-registered, calculated, and achieved sample size, suggesting high adherence by researchers to their previously met decisions. Regarding sample size determination, however, <60% provided any information, around one-third provided a separate paragraph featuring some of the most important SSP determinants, and only one in ten studies provided full information for comprehensive SSP. The limited predictive value of study design for actual sample size was found in a regression estimating the impact of SSP determinants together with selected study context variables, with the latter leading to substantial increases in sample size.

With a median sample size of 106 patients per study, the achieved sample size was comparable to earlier findings. For example, a survey in response to recommendations of the *APA Task Force on Statistical Inference* investigated changes in sample size over the past 30 years. Findings revealed a median sample size of *N* = 107 for studies published in 2006 in the *Journal of Abnormal Psychology* (Marszalek, Barber, Kohlhart, & Cooper, 2011). Since a significant increase in sample size only was observed from 1977 to 1996, the authors concluded that sample size remained rather constant over time—which also seems to apply to other types of clinical studies published in leading clinical psychology journals (Reardon, Smack, Herzhoff, & Tackett, 2019). Furthermore, a comprehensive review of studies registered in ClinicalTrials.gov found a medium sample size of only 61 participants (Califf et al., 2012). Although this discrepancy appears considerable (43%), the high variance between studies suggests no meaningful difference to the present investigation. Findings, therefore, support the interpretation of moderate and rather stable sample sizes.

According to standard power calculation software (e.g. G*Power; Faul et al., 2009), current RCTs for depression, therefore, are sufficiently powered to detect treatment effects of *d* = 0.5 using simple comparison (e.g. independent *t* test or between-group factor). Simultaneously, those numbers impede relevant analyses for the further advancement of the field, such as investigating therapy mechanisms or differential treatment effects. Additionally, many studies fail to provide a rationale for determining the expected treatment effect, while effects clearly differ as a function of study design (e.g. type of control group) and study context (e.g. efficacy *v.* effectiveness trial) (Cuijpers, van Straten, Bohlmeijer, Hollon, & Andersson, 2010; Kraemer, Mintz, Noda, Tinklenberg, & Yesavage, 2006). At this, the relevancy of study context is also being highlighted by a clear excess in the patient numbers for digital interventions compared to face-to-face treatment.

Study context appears reasonably impactful and can help explain the rather stable sample sizes. We estimated the proportions to which study context and study design (SSP determinants) influence sample size. Linear regression revealed that study context accounted for 81.1% of variance, leaving only 18.9% attributable to SSP determinants (cf. Fig. 2). This means that in the overall picture study context has been identified as a crucial factor in sample size determination. This proportion shifted to 58 and 42% of explained variance for those studies that featured SSP in their articles. Despite more balanced proportions in this subgroup, this pattern still underlines the relevancy of the study context even for RCTs featuring SSP. It, therefore, seems advisable to pay attention to restrictions arising from the study context. For example, more emphasis should be placed on intense assessment to increase statistical power, which we address later in this section. Another context factor concerns the distinction between efficacy and effectiveness studies. There exists solid evidence for higher treatment effects in efficacy trials (Cuijpers et al., 2010; Kraemer et al., 2006). However, many studies fail to mention this aspect when providing a rationale for their proposed treatment effect. Finally, some guidelines suggest to incorporate budget considerations into SSP (Bell, 2018). Representing a limitation to our findings, the presented proportions should be interpreted as approximations depending on the specified regression model. Additionally, sample size limits the information about single determinants of the regression model, which is why we abstain from interpreting single predictors in the model.

Considering the rather stable trial numbers of the last decades, the clear excess in achieved sample size of digital interventions (around 80%) appears particularly meaningful. Almost all interventions were designed as guided Internet-based treatment, which has been found effective for many common mental health disorders, and which was on par with face-to-face treatment in a recent meta-analysis (Carlbring, Andersson, Cuijpers, Riper, & Hedman-Lagerlöf, 2018). Together with other advantages, such as standardized and efficient treatment provision, digital interventions can be regarded a statistically powerful vehicle in the toolbox of contemporary psychiatric research.

As a related aspect, the practical costs of automatized intense assessment are decreasing, as so-called blended interventions are increasingly being tested or implemented into psychiatric care (Kooistra et al., 2019; Lutz, Rubel, Schwartz, Schilling, & Deisenhofer, 2019). In short, blended therapy can be regarded as computer-supported and app-supported face-to-face treatment, which has been tested for individual and group treatment of common mental health disorders (Erbe, Eichert, Riper, & Ebert, 2017; Schuster et al., 2019). The magnitude of expectable gains in statistical power due to automatized intense assessment is considerable and could help to mitigate the current situation without necessarily increasing sample sizes—that are frequently being limited by restricted resources. For example, a hypothetical RCT with point assessments of psychopathology (pre–post assessment by questionnaire) would require 90 patients, but 8 (bi-) weekly assessments during the active trial phase reduce this number to 42–50 patients. At this, short pre–post ecological momentary assessment (EMA) can offer further choices for study design (Schuster et al., 2020).

Regarding sample size in the context of prospective study planning, present data indicate that researchers adhered in a striking manner to the specifications made during trial pre-registration. This is reflected by very high correlations between planned, required, and achieved sample size. Additionally, many studies pre-registered their trial, which probably reflects a general trend towards pre-registration (Nosek & Lindsay, 2018; Scott, Rucklidge, & Mulder, 2015). For RCTs in clinical psychology, lower rates have been reported until recently (Cybulski, Mayo-Wilson, & Grant, 2016; Scott et al., 2015), suggesting progress in the practice of prospective study registration.

For scholarly SSP (cf. CONSORT 2010 guidelines in Box 1), however, a good part of the road to rigor still lies ahead. Only one-third provided information on basic SSP determinants, and only one in ten studies provided sufficient information for comprehensible SSP. It, therefore, remains unclear, how pre-registered sample sizes and sample sizes in SSP were exactly determined. For example, effect sizes for SSP for the wider field of psychology usually follow a more ample distribution (Kenny & Judd, 2019; Kühberger, Fritz, & Scherndl, 2014), as one would expect from a complex situation. The present analysis, however, revealed a narrow peak of expected effect sizes exactly at *d* = 0.5 (cf. Appendix 1). Additionally, practically all studies with more than two trial arms (e.g. two active and one passive group) featured only one effect size for SSP. Furthermore, less than one-third provided a rationale for how the proposed effect size was determined. These findings fit Cohen's speculation that ‘*low level of consciousness about effect size*’ might contribute to the problem (Cohen, 1992; Maxwell, 2004), and they also fit with a related phenomenon previously described as *sample size samba* (Schulz & Grimes, 2005).

With regard to the limitations of the reported findings, the following considerations should be taken into account. The sample size of the studies under investigation was subject to wide variation, including small feasibility studies and large multicenter trials. In view of this fluctuation, more extensive meta-analyses could provide additional results. For example, it would have been interesting to investigate SSP in specific study clusters. Given the assumption that Internet interventions are less restricted by study context, it would also have been interesting to investigate SSP in this group more closely. Regarding the conducted regression analyses, it should be noted that the proportion of explainable variance depends on the variables included in the model. Here, central study design and study context variables were included, but other parameters could be added as well. Importantly, as the present sample was restricted to 78 studies, we tried to abstain from interpreting single predictor variables of the multiple regression due to the risk of fluctuation. Instead, statistical power is sufficient to interpret the reported blocks of study design and study context. Concerning the generalizability of the reported findings, it can be assumed that reported patterns are likely not restricted to depression research. At the same time, further investigations are needed to draw safe conclusions. For this purpose, revision of SSP practice of RCTs for other common mental health disorders would be advisable.

## Conclusions

Although SSP is central to the planning of efficient trials, the majority of RCTs for the treatment of depression use no or limited SSP. While the case numbers of pre-registered studies have been achieved, the factors for calculating the required sample size remain unclear. The comparison of study design and study context showed a high relevance of study context, which probably is related to the rather stable trial numbers of the last decades. Here, the advancing developments in the field of digital psychiatry can provide feasible strategies (e.g. intense assessment and Internet-based treatment) to improve the situation.

## Supplementary material

For supplementary material accompanying this paper visit https://doi.org/10.1017/S003329172100129X. click here to view supplementary material
