# Supplementary material for: Sample size, sample size planning, and the impact of study context: systematic review and recommendations by the example of psychological depression treatment
Source: Psychol Med. 2021 Apr 21;51(6):902–8. doi: 10.1017/S003329172100129X (PMC8161431; doi:10.1017/S003329172100129X)
Supplement: Supplementary file 1 [file S003329172100129Xsup001.docx]

**Appendix 1**

**Recommendations for the SSP process**

This section provides suggestions for optimal SSP. As a general rule, researchers should provide as much information as possible, preferably in a separate paragraph in the methods section. In particular, researchers should:

- Provide all information presented in *Box 1* of the introduction.
- Assure that the specified SSP model fits the planned statistical analysis. For example, do not use *t*-tests during SSP if the planned analysis is based on interactions (e.g., ANCOVA or hierarchical models).
- Specify alpha and beta level, including information on one- or two-sided testing.
- Provide a clear rationale for the expected treatment effect (preferably based on meta-analytic evidence).
- Specify correlation among repeated measures as it influences estimates. Approximations can be found in literature, or can be derived from data sets. Bear in mind that treatment and larger time intervals (e.g. follow-up) will reduce this value.
- Provide evidence for expected dropout rates - again, preferably on basis of meta-analytic evidence (cf. *Table 2*).
- Bear in mind that you may rely on posthoc tests or relevant subgroup analyses. At this, consider their distribution as subgroups will not always be balanced (e.g. gender). Distributions are also relevant across multi-center research sites [43].

**Recommendations for efficient SSP**

This paragraph provides considerations for the design of future studies. As a general strategy based on the present evaluation, more emphasis should be placed on intense assessment as it yields the advantages of increased statistical power together with the choice of studying therapy process in more detail:

- Regard study context in SSP. For example, larger effects can arguably be found in efficacy compared to effectiveness trials [33]. Conversely, be aware of risks when assessing effect size through pilot studies [34].
- Whenever study context restricts achievable sample size for addressing important research questions, high quality study design (e.g. high treatment fidelity, additional process analyses) together with a neat pre-registration process can be help to demonstrate rigor.
- In times of increased automatization, consider intense assessment during the active period of a trial to booster power. While occasionally added assessments lead to marginal improvements, intense assessment will result in considerable reductions of required sample size [23].
- Do not substitute complex study designs by simple statistical models in SSP - e.g. do not calculate *t*-tests when the study design is factorial as you might sacrifice the advantage of repeated assessments. Instructions for more complex models (e.g. hierarchical models) can be found online [37]. Online courses for complex SSP are available (e.g. coursera.org).
- Be aware of adaptive clinical trial designs that allow sample size reassessment [38-41]. Describe the termination mechanism in detail during trial pre-registration. Bayesian approaches exist as well.
- Some SSP calculators offer the choice between specifying “full model” or “interaction only”. Several blogs explain their difference in an easy to understand way [42]. Interaction only can sometimes result in too low estimates. For comparison, power curves based on computer simulation are provided in Appendix 1, and power tables for RCTs can be found online.
- Consider general strategies to optimize power, such as preventing dropout, maintaining program integrity, covariate adjustment (e.g. baseline), or imputation techniques.

Rating

| 1. Design | 1 = a vs b  2 = a vs b vs c  3 = a vs b vs c vs d  4 = else:___ |
| --- | --- |
| 1. Comparisson | 1 = active (unspecified)  2 = active (care / treatment as usual)  3 = active (minimal support)  5 = passive (unspecified)  6 = passive (waitlist) |
| 1. Sample size | (according to CONSORT flow-chart) |
| 1. A priori power analysis provided? | 1 = yes, separate paragraph  2 = yes, provided  3 = no |
| 1. Comments1: |  |
| 1. Study context? | 1 = effectiveness study  2 = efficacy study |
| 1. Analysis priciple? | 1 = ITT (intention-to-treat)  2 = PP (per protocol) |
| 1. Outcome identical to study protocol / pre-registration? | 1 = yes  2 = no  n.a. = not applicable |
| 1. Expected effect size (in Cohen’s d) |  |
| 1. Rationale for expected effect provided? | 1 = meta-analytic evidence or comparable  2 = several studies  3 = in house studies  4 = none |
| 1. Comments 2: |  |
| 1. Alpha level |  |
| 1. Power (Beta-level, a-priori) |  |
| 1. Correlation of repeated measures |  |
| 1. Adjustment for | 1 = Drop-out  2 = Missing-data handling (e.g. MI)  3 = else _______________  4 = none |
| 1. Drop out | (according to CONSORT flow-chart) |
| 1. Software for power calculation | 1 = G*Power  2 = else ______________  3 = none |
| 1. Statistical test in power calculation | 1 = ANOVA  2 = ANCOVA  3 = Regression  4 = Chi 2  5 = t-Test  6 = LMM mixed models  7 = else _____________ |
| 1. Comments 3: |  |
| 1. Calculated sample size |  |
| 1. Posterior power (if applicable) |  |
| 1. Statistical test (actually applied) | 1 = ANOVA  2 = ANCOVA  3 = Regression  4 = Chi 2  5 = t-Test  6 = LMM mixed models  7 = else _____________ |
| 1. Primary outcome signifikant? | 1 = yes  2 = no |
| 1. Statistical value | (T= ___ or F=___) |
| 1. Reported effect size (primary outcome) |  |
| 1. Interpretation of results (does interpretation fit results)? | [insert text + comments] |
| 1. How is statistical power being discussed? | [insert text + comments] |
| 1. Comments 4: |  |
| 1. General comments: |  |

All missing = 999; n.a. = 888

Examples for comments 1 [2, 3, 4]:

**Statistical power curves based on computer simulation**

Estimated power for ANOVA, ANCOVA, and short pre-to-post EMA for frequent sample sizes. Simulation parameters were based on several empirical studies. 4000 virtual studies (N=50-200) with 64 effect sizes were produced before the statistical test of interest was applied. Power was logged as the proportion of positive over all applied tests. Linear mixed models produced comparable outcomes. The full study with further simulations is provided [**here**](https://www.sciencedirect.com/science/article/pii/S221478291930082X#ec0005). Distance between solid and dashed blue lines: gains in power due to applying ANCOVA (with baseline level as covariate) instead of ANOVA. Distance between solid lines: gains in power due to multiple pre-post assessment.


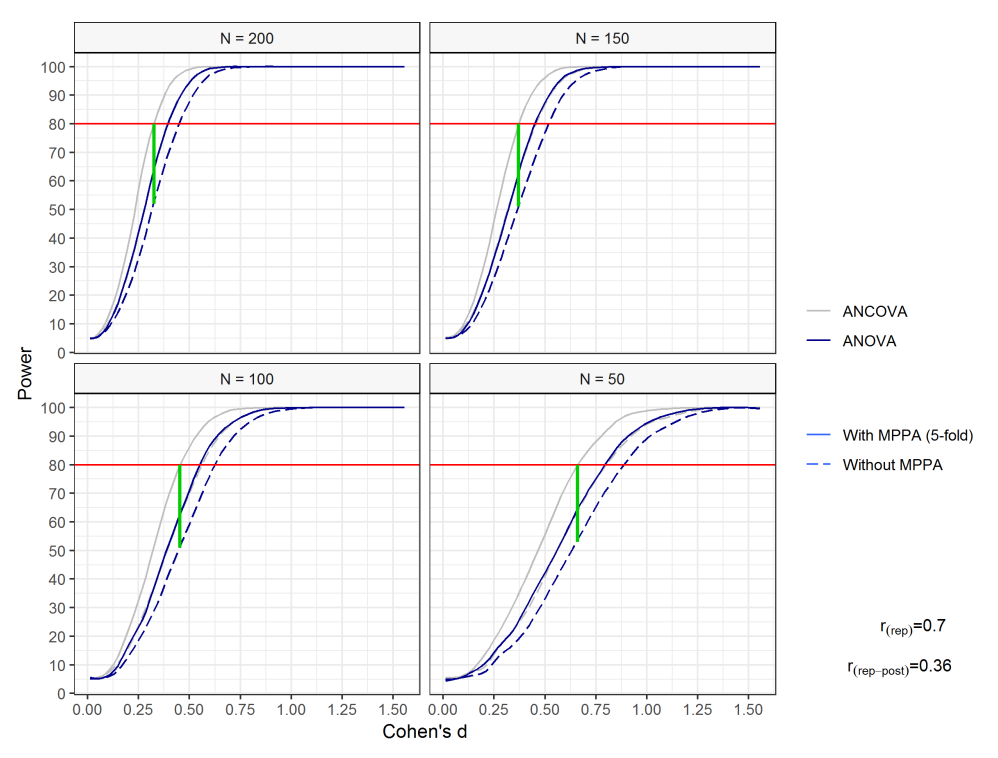


Note. ANCOVA = baseline covariate; MPPA = multiple pre-post-assessments (5-fold); r(rep) = correlation of repeated measures; r(pre-post) = correlation from pre- to post-assessment.
